# Individual and environmental risk factors for post-traumatic stress among hospital nurses after the 2024 Noto Peninsula earthquake in Japan

**DOI:** 10.3389/fpsyt.2025.1631694

**Published:** 2025-08-25

**Authors:** Naoki Furutani, Yuki Murata, Wataru Miwa, Masae Nakamura, Kakusho C. Nakajima-Ohyama

**Affiliations:** ^1^ Department of Psychiatry, Noto General Hospital, Nanao, Japan; ^2^ Department of Psychiatry, Nagoya City University East Medical Center, Nagoya, Japan; ^3^ Department of Psychiatry and Cognitive-Behavioral Medicine, Graduate School of Medical Sciences, Nagoya City University, Nagoya, Japan; ^4^ Department of Psychiatry and Behavioral Science, Graduate School of Medical Science, Kanazawa University, Kanazawa, Japan; ^5^ Department of Nursing, Noto General Hospital, Nanao, Japan; ^6^ Department of Neuropsychiatry, Nippon Medical School Hospital, Tokyo, Japan; ^7^ Department of Neuropsychiatry, Nippon Medical School Musashi Kosugi Hospital, Kawasaki, Japan

**Keywords:** post-traumatic stress disorder/symptoms (PTSD/PTSS), earthquake, older adults, Impact of Event Scale–Revised (IES-R), community damage

## Abstract

Although post-earthquake psychological distress arises from a complex interplay of personal vulnerabilities and environmental stressors, the pathways by which these factors interact remain underexplored. We surveyed 327 hospital nurses in Nanao City, Japan, approximately eight months after the magnitude-7.5 2024 Noto Peninsula earthquake; 224 complete responses were analyzed. Participants completed the Impact of Event Scale-Revised and a bespoke questionnaire assessing age, location during the earthquake, seven binary stress factors (home damage, relocation, community damage, change in co-residents, decline in family health, economic damage, earthquake-related sounds), and avoidant/emotion-focused coping. Analyses for each factor identified significant associations between IES-R scores and all stress factors except location during the earthquake and change in co-residents. ANCOVA adjusting for all predictors retained community damage, family health decline, economic damage, and coping as significant. Path analysis revealed two robust indirect pathways from age to distress: “age – community damage – IES-R” and “age – home damage – economic damage – IES-R”, plus a direct “age – intrusion” path. Decline in family health also influenced distress indirectly via economic loss. These findings demonstrate that older nurses’ elevated PTSS risk operates largely through greater exposure to specific disaster-related hardships, rather than age per se. Interventions should therefore combine individual support (e.g., coping skills, family health monitoring) with community-level recovery (e.g., infrastructure repair, social cohesion) to mitigate long-term mental-health impacts, especially among older adults.

## 1 Introduction

On January 1, 2024, a magnitude 7.5 earthquake (the 2024 Noto Peninsula Earthquake) struck the central-northern region of Japan, registering a maximum seismic intensity of 7 on the Japan Meteorological Agency (JMA) scale. This area had previously experienced a magnitude 6.9 earthquake with a maximum seismic intensity of 6-upper in 2007 (1). Nanao City, located in mid-peninsula, recorded a seismic intensity of 6-upper, sustaining the second-worst damage after the northern Noto area, which reached intensity 7. At Noto General Hospital, a month-long water outage prevented normal handwashing and patient care, creating unsanitary conditions and forcing staff to rely on alcohol-based disinfectants, and several clinical tests were halted for weeks. In addition to these harsh working conditions, staff members faced severe daily-life restrictions (even after their night shifts), such as driving over several hours for supplies and needing to use warmed purchased water for bathing over a three-month period. Road collapses and suspended public transportation were common, and both essential materials and repair workers were scarce. As of January 2025, some areas remain unstable due to the peninsula’s limited accessibility. At that time, the earthquake had led to 1,764 casualties overall, including 498 fatalities, with Nanao City alone reporting 79 casualties (42 fatalities). Across the Noto Peninsula, 83,299 houses were damaged, including 6,083 with “major damage,” defined in Japan as partial or complete collapse or structural damage affecting ≥50% of a house. Of these, 16,524 were in Nanao City, where 513 were classified as major damage. Although Nanao City’s losses were moderate compared to those in northern Noto, many residents experienced significant psychological distress resembling post-traumatic stress symptoms (PTSS). One outpatient described vivid, terror-filled recollections that have compelled them to continue sleeping near the front door even now, highlighting the disaster’s enduring impact.

Earthquakes can trigger overlapping stressors, ultimately giving rise to diverse psychological responses such as post-traumatic stress disorder or partial symptoms thereof (PTSD/PTSS) and depression (2, 3). In a ten-year longitudinal study, van den Berg et al. (4) examined trauma-related characteristics and found that the overall severity of these characteristics predicted PTSS at 18 months and again at 4 years post-disaster, even though no individual type of event (e.g., entrapment, injury, or bereavement) was a significant predictor on its own. However, by the 10-year follow-up this predictive effect had disappeared. The authors noted as a limitation that they had not assessed post-trauma factors such as coping strategies or social support, suggesting that these might supplant trauma-related characteristics as key predictors in the mid- to long-term.

The prevalence and severity of these conditions are influenced by multiple contributing factors beyond trauma exposure alone. In a meta-analysis of 52 observational studies from 1980 to 2016, Tang et al. (5) identified several risk factors, including personal characteristics (female gender, low education, prior trauma, low socioeconomic status), trauma-related characteristics (being trapped, experiencing fear or injury, or bereavement), and post-trauma characteristics (low social support, unemployment, property loss, or house damage). Additional studies have suggested that older adults (6–11), children and adolescents (3, 12), displacement (8, 9, 13–17), and avoidant/emotion-focused coping styles (13, 18) may further elevate psychological risk. Healthcare professionals in particular may face heightened stress because they must continue providing care even under compromised conditions and are often unable to take time off due to staffing shortages or ongoing patient needs, compounding the mental health burden of the disaster experience (19, 20). Taken together, this body of evidence underscores the multifactorial nature of post-earthquake stress symptoms, shaped by both contextual and personal factors, which is the focus of our study.

When considering the effects of multiple predictors in this way, univariate analyses, which cannot adjust for the influence of other variables, are often supplemented by multivariate methods such as analysis of covariance (ANCOVA) or multiple regression, both of which can handle several predictors simultaneously. However, these multivariate approaches treat all variables at the same hierarchical level and therefore cannot decompose and test causal pathways (direct versus indirect effects), potentially underestimating or omitting the influence of certain factors. Most of the previous studies introduced earlier are either collections of univariate analyses or regression and analysis of variance methods that analyze multiple variables on an equal footing. Ganime Can Gür (13) and Jiuping Xu et al. (18) employed hierarchical multiple regression, a technique in which predictors are entered in blocks by theoretical hierarchy so that residual variance not explained in earlier blocks is accounted for in subsequent blocks. Newnham et al. (3) used a latent growth curve model, which is designed to capture change over time rather than to detect indirect effects. We hypothesized that personal characteristics may influence post-trauma characteristics through complex interdependencies that ANCOVA alone cannot capture. Although our design is cross-sectional and cannot establish definitive causality, we conducted path analysis to define and quantify indirect pathways, thereby accounting for hierarchical interdependencies among predictors.

This study focuses on hospital nurses and aims to identify individual vulnerabilities and post-trauma stress factors related to symptom severity using the Impact of Event Scale–Revised (IES-R) alongside a bespoke questionnaire. By examining how personal predispositions and post-trauma conditions interact to sustain or perpetuate these stress symptoms, we seek to offer actionable insights for disaster mental health planning, particularly in contexts where physical infrastructure and daily necessities remain disrupted over prolonged periods.

## 2 Materials and methods

### 2.1 Participants

A total of 327 nurses working at Noto General Hospital (Nanao, Japan) were surveyed for PTSS and various factors using the Japanese-language version of the Impact of Event Scale–Revised (IES-R) (21) and an original Japanese questionnaire, respectively, approximately eight months after the main shock. This study was conducted in accordance with the ethical principles outlined in the Declaration of Helsinki and was approved by the ethics committee of Noto General Hospital. All participants provided informed consent with full assurance of anonymity.

### 2.2 Questionnaire items and variable selection

The Japanese version of IES-R comprises 22 items rated on a 5-point Likert scale (0–4), yielding a total score range of 0–88. The scale demonstrated high internal consistency (Cronbach’s α = 0.92 – 0.95) and good convergent validity (21). It can be subdivided into three subscales corresponding to the three PTSD symptom clusters: intrusion, avoidance, and hyperarousal.

In order to elucidate individual and environmental risk factors, we developed an original questionnaire for this study (**Table 1**). To enhance participant anonymity, respondents selected from predefined categories rather than reporting exact values: age was grouped as 20s, 30s, 40s, or 50 and above; and years of nursing experience were grouped as 1–2, 3–5, 6–10, 11–20, 21–30, or over 30. We also collected gender (male/female), location during the earthquake (inside or outside the hospital), and overall damage via a subjective, three-level self-assessment of household, property, or personal damage (“none,” “mild,” or “severe”). Our hospital was located approximately 40 km from the epicenter, so extremely severe exposures, such as being trapped under collapsed structures, sustaining serious physical injury, or witnessing death, were rare in our sample, and to limit respondent burden and maximize completion rates, we did not collect detailed trauma-event histories (e.g., entrapment, injury, or fatality exposure). Prior research has shown that indirect proxies of disaster exposure (e.g., home loss, community destruction, decline in family health, economic damage) correlate strongly with both the intensity of the traumatic experience and subsequent PTSS severity (22–24). We therefore assessed the presence or absence of seven specific stress factors: home damage, relocation of residence (e.g., evacuation to shelters, temporary housing, or moving), community damage (e.g., destruction of nearby houses or roads), change in co-residents (e.g., separation from or addition of family members), decline in family health, economic damage (e.g., job loss of a family member, significant repair costs), and earthquake-related sounds (e.g., emergency alerts, rumbling noises). Participants were also asked whether they engaged in avoidant/emotion-focused stress coping behaviors (Yes/No), which, in contrast to problem-focused behaviors, involve thinking more positively or taking mental breaks (25). Hereafter, avoidant/emotion-focused stress coping behaviors are referred to simply as “stress coping.” Among the 238 respondents, 224 provided complete and valid answers for every item and were included in the subsequent analyses. Their demographic data are summarized in **Table 2**.

**Table 1:** English translation of the original questionnaire items.

Question	Options
Q1. Personal information [Single choice]	
Age (as of now)	20s; 30s; 40s; 50 and above
Gender	Male; Female
Years of Nursing Experience (as of now)	1–2 years; 3–5 years; 6–10 years; 11–20 years; 21–30 years; 31 years or more
Location during the Earthquake	Inside the hospital; Outside the hospital
Overall Damage Severity (home/property/personal)	None; Mild; Severe
Q2: Stressors since the earthquake [Multiple choice]	
	Home damage
	Change of residence (e.g., shelter, temporary housing, moving, car living)
	Community damage (e.g., local houses or roads)
	Change in co-residents (e.g., separation from or addition of family members)
	Decline in family health
	Economic damage (e.g., job loss of a family member, significant repair costs)
	Earthquake-related sounds (e.g., emergency alerts, rumbling noises)
Q3: Stress coping behaviors [Single choice]	
Have you engaged in any avoidant/emotion-focused coping behaviors since the earthquake?	Yes; No

**Table 2:** Demographic characteristics of the participants (n=224) and their IES-R total scores.

Category	N	Percent [%]	IES-R total score [mean ± SD]
All participants	224	100.00	14.01 ± 13.55
Age [years]			
20 - 29	29	12.95	12.66 ± 15.48
30 - 39	48	21.43	10.17 ± 10.05
40 - 49	73	32.59	12.74 ± 11.54
≥ 50	74	33.04	18.30 ± 15.54
Gender			
Male	21	9.38	8.48 ± 9.86
Female	203	90.62	14.59 ± 13.76
Nursing experience [years]			
1 - 2	10	4.46	13.20 ± 21.94
3 - 5	15	6.70	12.47 ± 11.92
6 - 10	24	10.71	8.58 ± 8.78
11 - 20	56	25.00	12.12 ± 11.56
21 - 30	63	28.12	15.38 ± 14.07
≥ 31	56	25.00	17.25 ± 14.49
Location during earthquake			
In-hospital	47	20.98	16.70 ± 17.20
Out-hospital	177	79.02	13.30 ± 12.36
Overall damage			
No	12	5.36	8.42 ± 10.66
Mild	148	66.07	11.45 ± 11.48
Severe	64	28.57	21.00 ± 15.85
Home damage			
No	107	47.77	10.50 ± 10.65
Yes	117	52.23	17.23 ± 15.07
Relocation of residence			
No	203	90.62	13.29 ± 13.11
Yes	21	9.38	21.05 ± 15.86
Community damage			
No	112	50.00	10.53 ± 10.52
Yes	112	50.00	17.50 ± 15.28
Change in co-residents			
No	208	92.86	13.70 ± 13.46
Yes	16	7.14	18.12 ± 14.41
Decline in family health			
No	176	78.57	11.58 ± 12.11
Yes	48	21.43	22.94 ± 14.87
Economic damage			
No	178	79.46	11.85 ± 11.93
Yes	46	20.54	22.37 ± 16.12
Earthquake-related sounds			
No	79	35.27	10.91 ± 13.00
Yes	145	64.73	15.70 ± 13.58
Stress coping			
No	187	83.48	12.63 ± 13.16
Yes	37	16.52	21.00 ± 13.49

Because only a small number of male nurses participated, “gender” was excluded from further analysis. Likewise, “nursing experience” was considered redundant with “age” (Spearman’s ρ = 0.88; see S**upplementary Table 1** for the full correlation matrix) and was therefore excluded. The variable “overall damage” was also excluded because it represents a composite of the individual stress factors. Consequently, the final analysis focused on ten items: “age,” “location during the earthquake,” “home damage,” “relocation of residence,” “community damage,” “change in co-residents,” “decline in family health,” “economic damage,” “earthquake-related sounds,” and “stress coping.”

### 2.3 Statistical analysis

Next, we explored the relationships between various questionnaire items and IES-R total scores. In all analyses, the IES-R total score or subscale score served as the dependent variable, and all other questionnaire items were treated as independent predictors. For all analyses, independent variables were coded as follows: age was grouped into four categories (20s, 30s, 40s, 50 and above) and treated as an ordinal scale; all other items were binary or nominal and analyzed as categorical predictors. First, we examined bivariate associations: age was assessed via Spearman’s rank correlation, and all other dichotomous items (Yes/No or A/B) were compared using the Mann–Whitney U test, owing to non-normal score distributions (**Supplementary Figure 1**). To evaluate the combined influence of multiple predictors, we then performed an ANCOVA, which estimates each factor’s main effect while statistically controlling for the others in a single linear model. Finally, we conducted path analysis to partition total effects into direct versus indirect pathways and formally test the fit of these hypothesized relationships. Path analysis was performed using the Wishart maximum likelihood method. Initial models included age and the nine stress factors; paths that were not significant were sequentially removed. Furthermore, when candidate paths between the ten factors were added one at a time and ranked by Δχ², the paths from home damage and from decline in family health to economic damage consistently ranked among the top candidates for the IES-R total score and each subscore. Therefore, these two paths were added to produce the final model. Indirect effects were formally tested via 2,000 bootstrap resamples, with 95% percentile confidence intervals. Statistical significance was set at p < 0.05. All statistical analyses were conducted using Python modules. Spearman’s rank correlation and the Mann–Whitney U test were performed using SciPy (26), and path analysis was conducted using Semopy (27).

## 3 Results

### 3.1 Univariate associations between stress factors and IES-R scores

**Figure 1:**
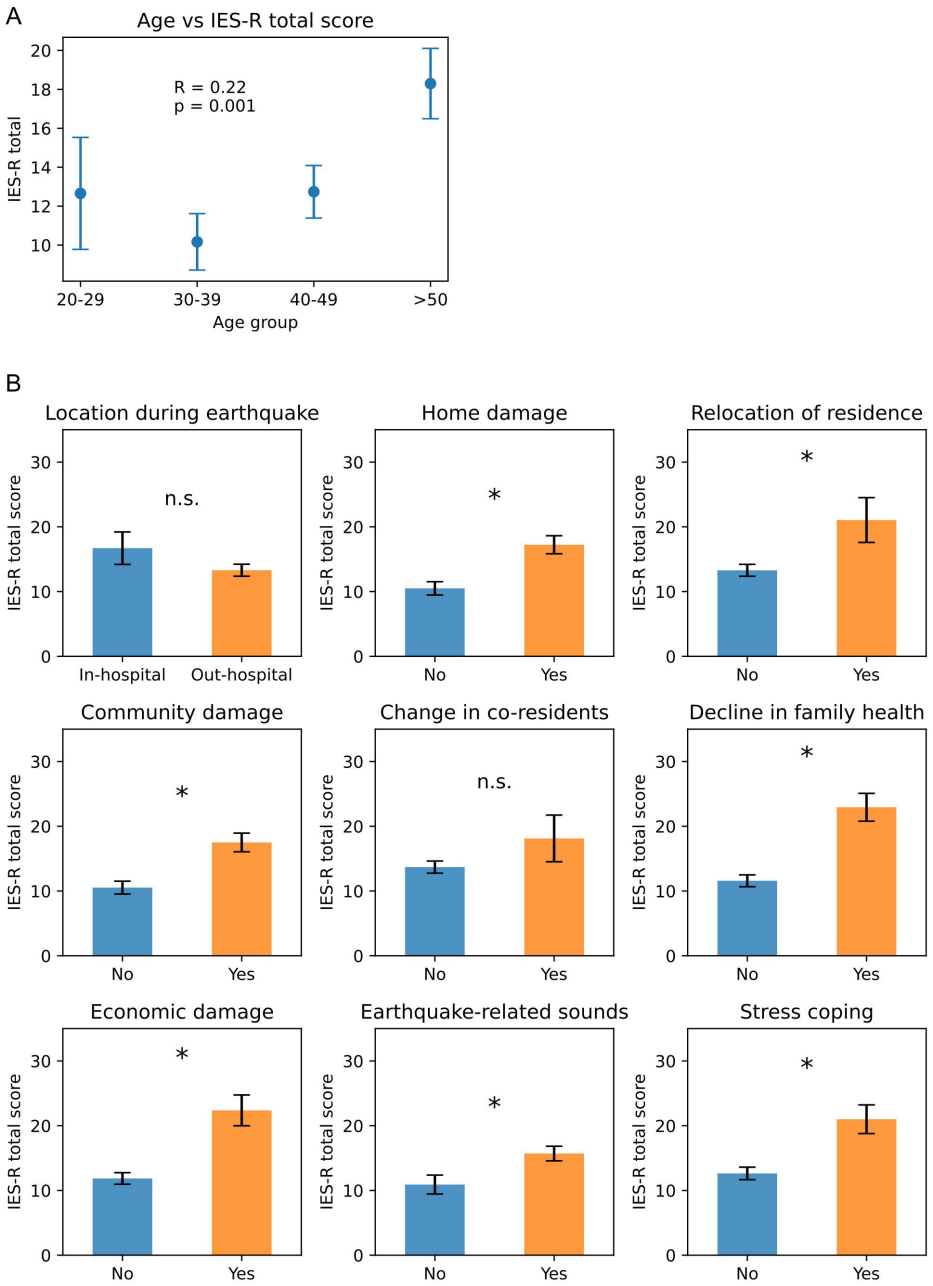
Individual analyses of each factor’s impact on IES-R total scores. **(A)** Relationship between age and total IES-R score (mean ± SD). R and p values indicate Spearman’s rank correlation. **(B)** Distribution of IES-R scores in participants without vs. with each stress factor. p values are from the Mann–Whitney U test (*p < 0.05; n.s., not significant).

Initially, we examined the relationship between each questionnaire item and the IES-R total score separately. “Age” was significantly correlated with IES-R (**Figure 1A**). Among the remaining nine factors, only “location during the earthquake” and “change in co-residents” showed no significant group differences; the other seven factors exhibited significant differences between the Yes and No groups (**Figure 1B**).

### 3.2 The combined effects of stress factors: ANCOVA

Next, an ANCOVA was performed to assess the overall impact of all questionnaire items on the IES-R (**Table 3**). Similar to the individual analyses, “community damage,” “decline in family health,” “economic damage,” and “stress coping” had significant effects. In contrast, several variables that were individually significant (“age,” “home damage,” “relocation of residence,” and “earthquake-related sounds”) did not remain significant in the ANCOVA.

**Table 3:** ANCOVA results for the impact of age and the various stress factors on IES-R total scores.

Variable	Coefficient	Std. error	t	p
Intercept	2.288	3.477	0.658	0.511
Age	0.119	0.081	1.467	0.144
Location during earthquake	-1.853	1.998	-0.928	0.355
Home damage	2.709	1.743	1.555	0.122
Relocation of residence	1.891	3.078	0.614	0.540
Community damage	3.925	1.676	2.342	0.020*
Change in co-residents	-2.895	3.452	-0.838	0.403
Decline in family health	7.377	2.119	3.481	0.001*
Economic damage	5.863	2.214	2.648	0.009*
Earthquake-related sounds	2.274	1.739	1.308	0.192
Stress coping	6.124	2.178	2.812	0.005*

*p < 0.05.

“In-hospital” is used as the reference in “Location during earthquake.”

### 3.3 Pathways of age-related effects on PTSS: path analysis

**Figure 2:**
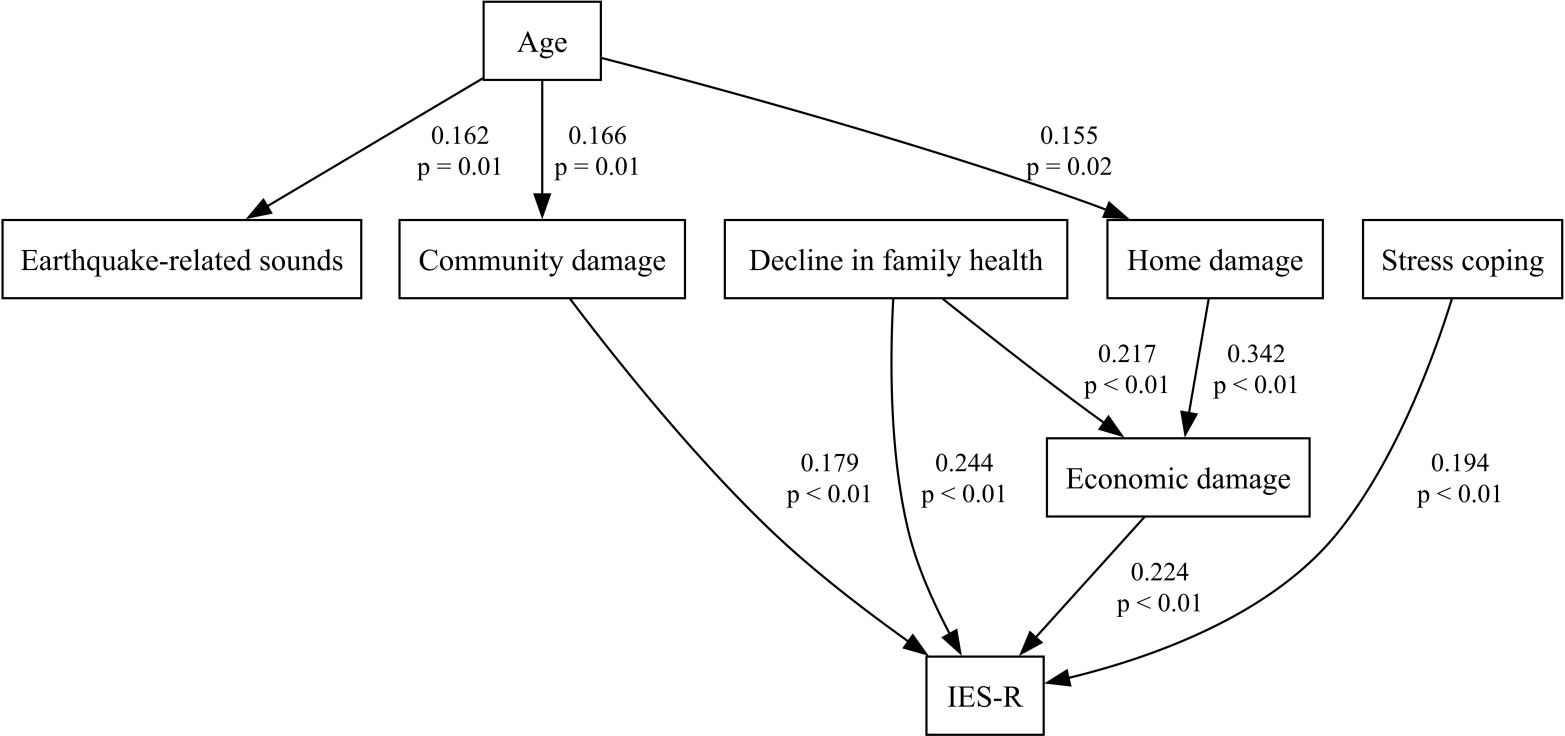
The final model of path analysis describing the relationships among age, each stress factor, and the IES-R total score. Numbers above each path indicate standardized coefficients; numbers below are p values.

**Figure 3:**
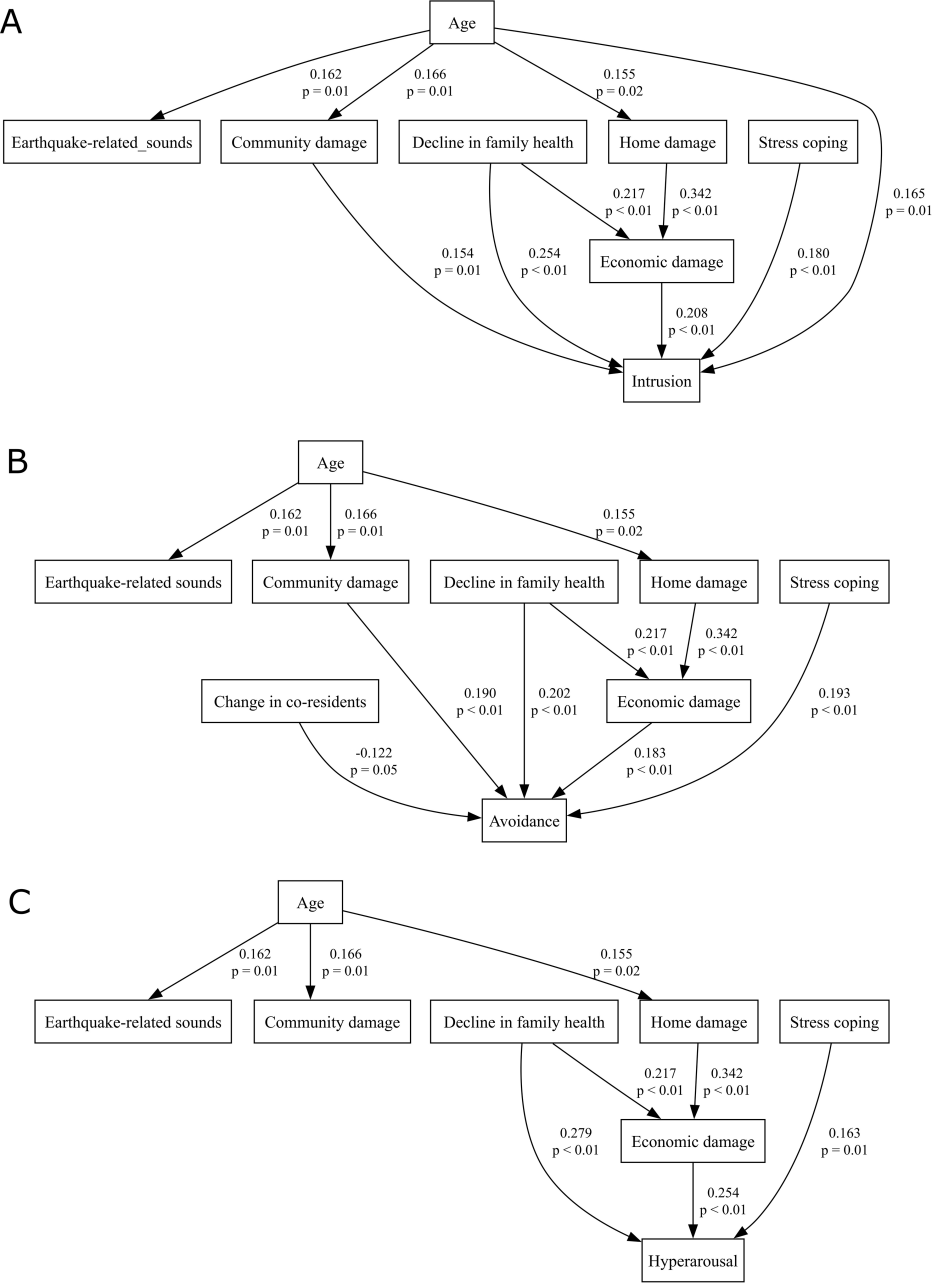
Path analysis models for the relationships among age, each stress factor, and the three IES-R subscores **(A)** intrusion, **(B)** avoidance, **(C)** hyperarousal. Numbers above each path indicate standardized coefficients; numbers below are p values.

Furthermore, to distinguish between direct and indirect effects, we conducted path analysis, which revealed two indirect pathways from age to PTSS via community damage and via a sequence from home damage to economic damage (Root Mean Square Error of Approximation (RMSEA) = 0.054, Comparative Fit Index (CFI) = 0.89) (**Figure 2**). All indirect paths were significant (95% CI for the standardized coefficient: Age–Community damage–IES-R, 0.0034–0.0650; Age–Home damage–Economic damage–IES-R, 0.0013–0.0283; Decline in family health–Economic damage–IES-R, 0.0132–0.1022). Further analyses of the three IES-R subscales (intrusion, avoidance, hyperarousal) identified largely similar pathways to the total score model, with some subscale-specific differences: a direct path from age to intrusion and a path from change in co-residents to avoidance were found, whereas the path from community damage to hyperarousal was no longer retained (RMSEAs of 0.050, 0.038, and 0.056, and CFIs of 0.92, 0.93, and 0.88, respectively) (**Figure 3**). All indirect paths were significant (95% CI for the standardized coefficient: Age–Community damage–Intrusion, 0.0038–0.0654; Age–Home damage–Economic damage–Intrusion, 0.0011–0.0285; Decline in family health–Economic damage–Intrusion, 0.0107–0.0973; Age–Community damage–Avoidance, 0.0032–0.0651; Age–Home damage–Economic damage–Avoidance, 0.0004–0.0248; Decline in family health–Economic damage–Avoidance, 0.0059–0.0868; Age–Home damage–Economic damage–Hyperarousal, 0.0008–0.0309; Decline in family health–Economic damage–Hyperarousal, 0.0136–0.1067).

## 4 Discussion

### 4.1 Interpretation of the impact of age and other stress factors on PTSS

In Nanao City, where our hospital is located, losses were moderate compared to northern Noto; however, a subset of hospital staff still reported PTSS. Even considering that the Japanese-language version of the IES-R uses a lower cutoff of 25 points (instead of the usual 33 points) and that our sample comprised hospital nurses rather than the general population, the mean ± SD IES-R score in our study was 14.01 ± 13.55 (**Table 2**), which is substantially lower than the 33.1 ± 17.4 reported after the 2008 Wenchuan Earthquake (magnitude 8.0) (28) and the 45.32 ± 18.75 observed following the 2023 Türkiye earthquakes (magnitudes 7.7 and 7.6) (13), suggesting less overall distress. However, our median IES-R score was 10 (IQR 4–21), which was comparable to the 9 (IQR 4–18) reported among nurses eight to nine months after the 2016 Kumamoto Earthquake (magnitudes 6.5 and 7.3, both with a maximum seismic intensity of 7) (29), indicating similar persistence of PTSS. In other words, stress levels in our study were on par with those seen after considerably stronger earthquakes.

Although our initial univariate analyses showed that age was a strong predictor of earthquake-related PTSS (**Figure 1**), its effect disappeared when all factors were entered together in an ANCOVA (**Table 3**). This pattern led us to propose that personal vulnerabilities like age may shape symptom severity indirectly through intermediate disaster‐related stressors. Indeed, path analysis revealed that age influenced IES-R scores indirectly via community damage and via a sequence from home damage to economic damage (**Figure 2**). Previous studies on age effects in earthquake-related stress have been inconsistent: some identify older age as a risk factor (6–11), while others find older adults to be more resilient or even protected (18, 30). Middle-aged groups have been linked to higher PTSD risk (31), yet some reports show lower suicide rates in this same demographic (32). Likewise, one study notes a linear rise in suicide risk from ages 15 to over 65 years (33), whereas another study points to greater vulnerability among the young (3). Even among adolescents and young adults, findings diverge: one study shows that lower age corresponds to higher mental risk (12), while others indicate that older adolescents experience greater stress (13, 34). Several studies report no clear relationship between age and post-earthquake stress (14, 15, 17, 35–37). These contradictions may be explained by variations in diagnostic criteria, analytic methods, and sample characteristics (5, 8, 38). In our data, older age raised vulnerability to community damage, home damage, and earthquake-related sounds; both community damage and consequent economic loss were linked to higher PTSS (**Figure 2**). The paths from age to both community damage and home damage may stem from the fact that older adults often live in neighborhoods with older, more disaster-prone infrastructure. Research also shows that mutual community support can alleviate stress among older adults (39), so this demographic may be especially sensitive to perceived community damage during recovery. Furthermore, age directly predicted intrusion, suggesting possible biological susceptibilities or other unmeasured stressors. For example, school closures and shortened hours forced younger nurses with children to leave early or take leave, thereby increasing the relative workload and stress borne by their older colleagues. Regardless of respondent age, a decline in family health both directly and indirectly (via economic loss) exacerbated PTSS. Because Nanao City has a high proportion of elderly residents, some who had previously managed on their own suffered health setbacks after the earthquake, making daily life more difficult. Simultaneously, parents of young children sometimes struggled when their children, frightened by aftershocks, refused to attend school or lost their appetite. These dynamics imply that family members’ ages—not only the respondent’s age—played an indirect role in shaping post-traumatic stress. Overall, our findings suggest that post-earthquake mental-health strategies should combine individual-level interventions with community-level recovery efforts, especially for vulnerable groups such as older adults and families with young children.

Interestingly, avoidant/emotion-focused stress coping was positively associated with PTSS. This seemingly contradictory result might reflect reverse causality, whereby individuals experiencing greater distress are more inclined to rely on avoidant/emotion-focused coping strategies. Additionally, as noted in the previous paragraph, avoidant coping may impair opportunities for fear memory extinction. Several studies of earthquake survivors, including those after the 2008 Wenchuan quake in China (18), the 2009 L’Aquila quake in Italy (36), and the 2023 Türkiye quake among youth (13), have found that problem-focused coping typically yields better mental-health outcomes than avoidant/emotion-focused approaches. Accordingly, in our path model the positive coefficient for avoidant/emotion-focused coping likely reflects its less adaptive role. Moreover, coping-style differences have been linked to mental-health risk particularly among women (18).

Furthermore, differences among the IES-R subscales, including intrusion, avoidance, and hyperarousal, may hold important clinical implications. As an indirect effect of age, economic damage amplified all PTSS, whereas stress arising from community damage manifested primarily as intrusion and avoidance symptoms rather than hyperarousal. Intrusion also exhibited a direct effect of age. Although earthquake-related sounds are thought to be most closely linked to intrusion, the direct influence of age on intrusion may have led to an underestimation of the effect of earthquake-related sounds on this symptom cluster. Change in co-residents was included in our questionnaire as a presumed stressor; paradoxically, it emerged as a factor mitigating avoidance, possibly because, by relocating away from trauma-related cues and environments, individuals no longer needed to engage in avoidance behaviors. The specific pattern of PTSS can provide clues to underlying physiological mechanisms. Each symptom cluster, intrusion, avoidance, and hyperarousal, shares common substrates of amygdala-centered fear-circuit hyperactivity and dysregulation of the prefrontal–hippocampal network (40), as well as HPA-axis imbalance, noradrenergic hyperactivity, and serotonergic hypofunction (41). In addition, distinct mechanistic biases have been reported for each cluster: intrusion is particularly associated with early overconsolidation of fear memories driven by amygdala hyperactivity and impaired hippocampal–prefrontal regulation (40, 42); avoidance reflects not only reduced opportunities for fear extinction due to limited trauma exposure but also transient activation of the amygdala, hippocampus, and medial prefrontal cortex that interferes with extinction learning (43), as well as dopamine-reward-circuit dysfunction resembling depressive features (40); hyperarousal is characterized by sympathetic overdrive mediated by brainstem-limbic vigilance systems (44). In our study, older age was directly associated with the emergence of intrusion symptoms (**Figure 3A**), suggesting that pharmacotherapies such as SSRIs, targeting the shared mechanism among all PTSS clusters, may be particularly important. Conversely, the indirect effects of aging appear to be context-dependent. For instance, because community damage was linked to avoidance as well as intrusion (**Figures 3A, B**), combining behavioral interventions such as exposure therapy may be warranted in such cases (43). Moreover, earthquake-related PTSD/PTSS and depression are closely tied to sleep disturbances (35, 45), and prolonged REM sleep fragmentation is a key predictor of PTSD onset (46). This may involve amygdala-, dopamine-, and serotonin-mediated processes implicated in REM induction (47, 48). These findings underscore the value of brain-function measurements, such as EEG, neuroimaging, or wearable sleep trackers, not only for early detection and intervention but also for guiding personalized treatments tailored to each individual’s specific symptom profile and underlying neurobiological mechanisms. Potential PTSD biomarkers extend beyond monoamines to include abnormalities in GABA, neuropeptides, BDNF, HPA-axis dysregulation, and inflammatory cytokines (49, 50). Blood testing at our hospital was briefly suspended, but EEG monitoring remained feasible, highlighting the need for various biomarker-based screenings in future earthquake scenarios.

Even areas not struck by a truly major disaster can still endure chronic stressors, frequent aftershocks, persistent community damage, family health problems, and economic hardship, and such long-term disruptions may give rise to PTSS. This observation aligns with previous work showing that psychological disturbances after earthquakes often persist for extended periods (3). Although fundamental evidence on PTSD’s underlying mechanisms remains scarcer than for depression or anxiety disorders, early, multifaceted interventions, including routine screening with self-report instruments and physiological biomarkers, hold promise for mitigating protracted stress.

### 4.2 Limitations

This study has some limitations. The primary limitation of this study is the lack of detailed trauma‐event histories. To maximize questionnaire completion rates, we intentionally kept the survey brief, omitting items on specific exposures (e.g., entrapment, injury, bereavement). However, because the IES-R correlates moderately with a broad range of psychological conditions beyond PTSS (51), it may have captured symptoms related to disorders other than PTSD/PTSS. In addition, depression, anxiety disorders, and other diagnoses can arise, each with different durations and contributing factors (3, 35). Moreover, our retrospective survey—conducted eight months after the earthquake—introduces potential recall bias, as participants’ memories of both exposures and symptoms may have faded or shifted over time. Future research should therefore employ multiple validated psychometric instruments alongside physiological assessments, and ideally collect data closer to the event (with follow-up waves), to better distinguish among diverse post-disaster mental-health outcomes and minimize recall bias.

Additionally, our sample comprised exclusively actively employed hospital nurses, predominantly female and under age 70, who may differ systematically from the wider survivor population. Healthcare workers face unique disaster-related stresses (e.g., overwork, resource scarcity, caregiving burdens) (19, 20) that limit generalizability. Moreover, by excluding non-employed or severely affected individuals, we may have underestimated symptom severity. All participants were also sufficiently healthy to remain employed, which could exclude those with more severe physical or mental health conditions. Replication in diverse demographic and occupational cohorts is needed.

The use of path analysis allowed us to model indirect pathways, but causal inferences remain tentative in a cross-sectional design. We did not predefine causal ordering among all stress factors, and residual confounding or collinearity, especially among co-occurring exposures, may bias estimates. In the previous section, we discussed only those effects that were significant in our path analysis; however, predictors that were significant in bivariate analyses but not in multivariate models should not be assumed unimportant, as their shared variance may still reflect meaningful relationships. Longitudinal studies with larger samples and latent-variable mediation models are required to confirm the direction and magnitude of the observed relationships.

All stress factors and outcomes were self-reported, raising the possibility that individual differences in stress sensitivity influenced responses. Additionally, we did not include objective damage assessments (e.g., structural surveys, medical records), which could strengthen validity. Future work should integrate self-report with objective indicators.

Despite these limitations, our findings offer novel insight into how demographic variables like age selectively shape the impact of specific disaster-related stressors. By identifying both direct and indirect pathways to PTSS, this study provides a foundation for targeted interventions at both the individual and community levels.

## Data Availability

The datasets presented in this article are not readily available because of the nature of the research, due to ethical reasons. Further inquiries can be directed to the corresponding author. Requests to access the datasets should be directed to furutaninaoki@gmail.com.
