# Supplementary material for: Individual and environmental risk factors for post-traumatic stress among hospital nurses after the 2024 Noto Peninsula earthquake in Japan
Source: Front Psychiatry. 2025 Aug 25;16:1631694. doi: 10.3389/fpsyt.2025.1631694 (PMC12415647; doi:10.3389/fpsyt.2025.1631694)
Supplement: Supplementary file 1 [file Supplementaryfile1.docx]

Supplementary Material

# Supplementary Tables


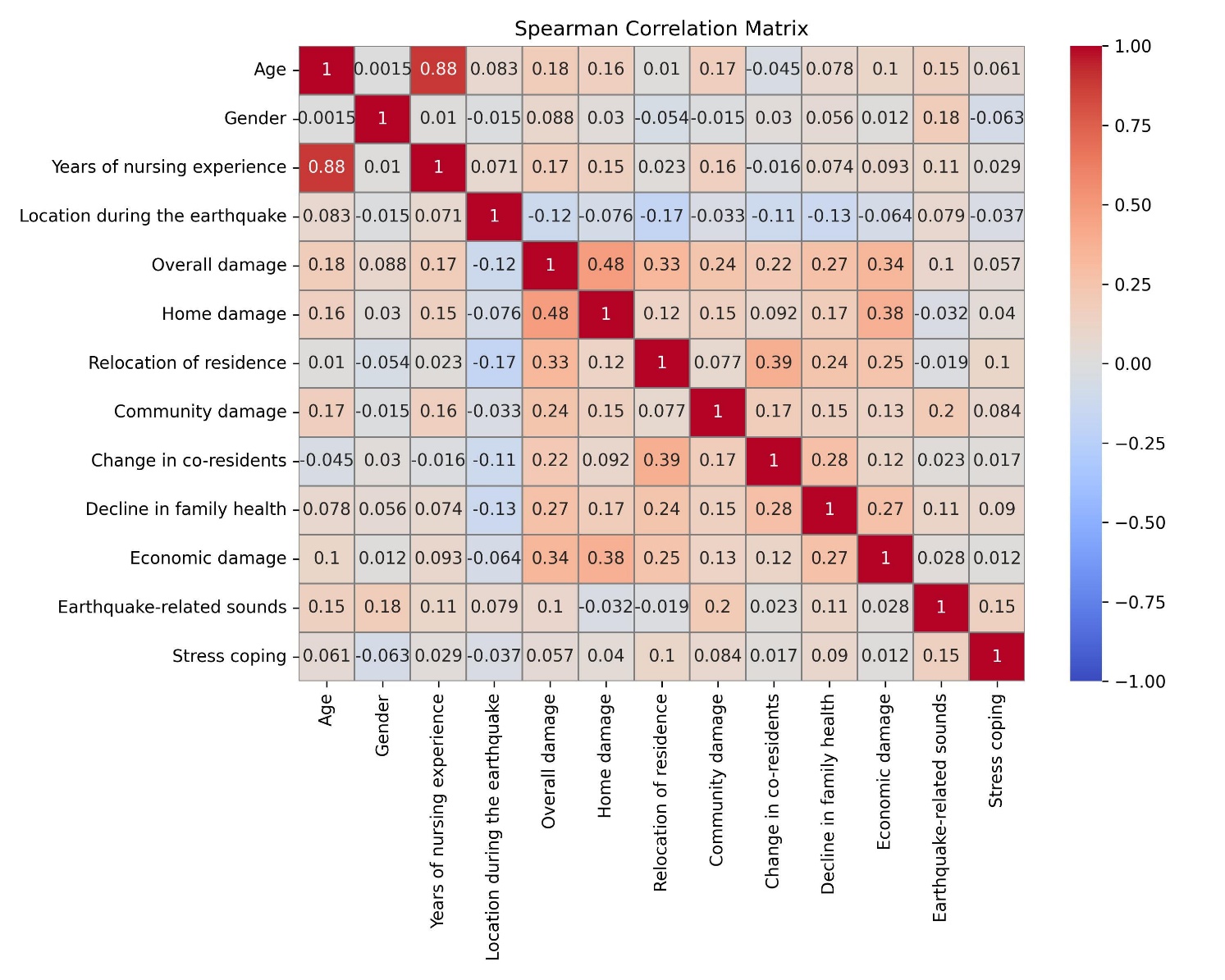


**Supplementary table 1.** Spearman’s rank correlation matrix for independent variables.

# Supplementary Figures


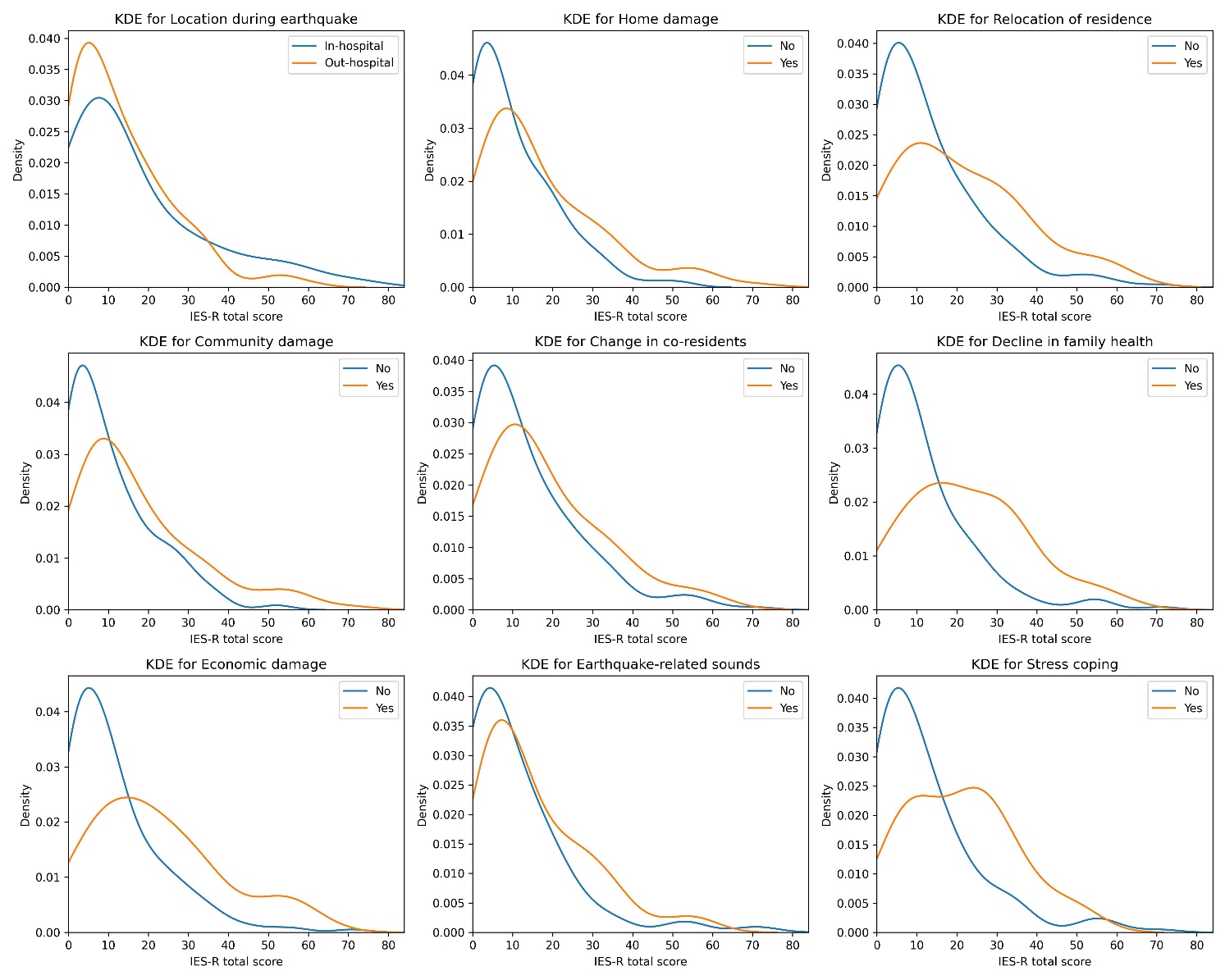


**Supplementary Figure 1.** Distribution of IES-R total scores for each stress factor (presence vs. absence).
